# Healthy aging and late-life depression in Europe: Does migration matter?

**DOI:** 10.3389/fmed.2022.866524

**Published:** 2022-11-07

**Authors:** Ivet Bayes Marin, Daniel Fernández, Jose Luis Ayuso-Mateos, Matilde Leonardi, Beata Tobiasz-Adamczyk, Seppo Koskinen, Albert Sanchez-Niubo, Paula Cristóbal-Narváez

**Affiliations:** ^1^Departament de Medicina, Facultat de Medicina i Ciències de la Salut–Campus Clínic, Universitat de Barcelona, Barcelona, Spain; ^2^Centro de Investigación Biomédica en Red de Salud Mental, Instituto de Salud Carlos III, Madrid, Spain; ^3^Serra Húnter fellow, Department of Statistics and Operations Research (DEIO), Universitat Politècnica de Catalunya BarcelonaTech, Barcelona, Spain; ^4^Institute of Mathematics of UPC–BarcelonaTech, Barcelona, Spain; ^5^Department of Psychiatry, Universidad Autónoma de Madrid, Madrid, Spain; ^6^Neurology, Public Health, Disability Unit–IRCCS Neurology Institute Besta, Milan, Italy; ^7^Department of Medical Sociology, Jagiellonian University Medical College, Kraków, Poland; ^8^Department of Public Health Solutions, National Institute for Health and Welfare (THL), Helsinki, Finland; ^9^Department of Social Psychology and Quantitative Psychology, University of Barcelona, Barcelona, Spain; ^10^Research, Innovation and Teaching Unit, Parc Sanitari Sant Joan de Déu, Barcelona, Spain

**Keywords:** late-life depression, healthy aging, migration, harmonized data, multivariate logistic regression

## Abstract

**Background:**

There is limited research examining the impact of risk and protective factors on late-life depression using large population-based datasets, particularly those examining differences among older migrants and non-migrants in Europe countries. Thus, the first aim was to analyze differences between migrants and non-migrants regarding socioeconomic status, depression, multimorbidity, healthy aging, and lifestyle behaviors. The second aim was to examine the impact of healthy aging on late-life depression in older migrants compared to their counterparts without a history of international migration in extensive and harmonized data from different population-based cohort studies.

**Materials and methods:**

We analyzed cross-sectional, predominantly nationally representative, community-based data from European participants in the Aging Trajectories of Health: Longitudinal Opportunities and Synergies (ATHLOS) cohort. The descriptive analyses included sociodemographic variables, somatic comorbidities, multimorbidity, healthy aging, and lifestyle behaviors according to migration status. The effects of these variables on late-life depression were examined in a multivariate logistic regression model, including migration status and years since migration as predictors.

**Results:**

Data of 122,571 individuals aged ≥ 50 years were analyzed, of which 11,799 (9.60%) were migrants. The descriptive analyses indicated that compared to non-migrants, migrants showed a higher prevalence of diabetes (25.6%), hypertension (38.0%), coronary artery disease (49.4%), stroke (4.9%), and depression (31.1%). Healthy aging was also better in non-migrants (51.7; SD = 9.7) than in migrants (39.6; SD = 18.2). The results of the logistic regression showed that migration status [OR = 1.231 (CIs: 0.914–1.547)] and increased number of years since migration in the host country [OR = 0.003 (CIs: 0.001–0.005)] were associated with greater levels of depressive symptoms. Concerning health variables, multimorbidity was associated with higher levels of depressive symptoms [OR = 0.244 (CIs: 0.211–0.278)], whereas better healthy aging was associated with fewer depressive symptoms [OR = -0.100 (CIs: -0.102 to -0.098)]. The interaction between migration and healthy aging status was also significant [OR. = -0.019 (CIs: -0.025 to -0.014)].

**Conclusion:**

Migrants reported higher risks for worse health outcomes compared to non-migrants. Significantly, worse healthy aging was associated with a greater risk of depressive symptoms in migrants than in non-migrants. Shedding light on migration and aging processes is essential for promoting a cross-cultural understanding of late-life depression in Europe.

## Introduction

In the 21st century, the dynamic process of globalization has implied an economic, technological, political, social and cultural interconnection of the entire globe, with a continuous migration of people across national borders in pursuit of better life chances. In the 50s and 60s, European history was characterized by more significant numbers of labor migrants moving from Southern to Northern parts of Europe, and, in the last decade, immigration to Europe from Non-European countries has rapidly increased. As a result, current European societies have to face new social and healthcare challenges with an increasingly aging and culturally diverse society (1).

The experience of migration impacts well-being and physical and mental health (2). A great body of data has indicated that several risk factors are associated with an increased risk for mental disorders in migrants, including individual characteristics (i.e., age, sex, world region of origin, and education), factors surrounding the migration process (i.e., reason for migrating, acculturation, and language proficiency) and post-migration stressors related to the social and occupational environment in the host country. Indeed, an increased risk for psychotic disorders, psychosomatic disorders, anxiety, and depression have been reported to be prevalent in some migrant populations (3). For instance, a recent systematic review and meta-analysis based on 25 studies showed that the aggregate prevalence of depression among international migrants was 15.6% across 20 different countries (4). Since migrants may be a vulnerable group in any population, more extensive studies are needed focusing on the potential risk of factors negatively affecting their mental health.

The aging process may pose additional burdens on migrants, increasing the risk for emotional problems. Converging evidence suggests that migrants may face greater challenges with age, such as ambivalence about returning to their country of origin, retirement, acculturation level of family support, lower income levels, and loss of social roles (5). Notably, due to their role and status in many societies, migrant women may be particularly vulnerable to increased risk for emotional problems, particularly in the elderly (6).

The study of the health of older migrants in Europe is one of the priorities of European healthcare systems and is extremely useful for policymakers attempting to promote health equity interventions (7). Previous research has shown that compared to non-migrants, older migrants in Europe tend to report worse health status, poorer functioning, more chronic physical conditions, and a higher prevalence of depression (6, 8–10). Importantly, depression in the elderly (11) is also associated with a greater risk of morbidity and suicide, poorer cognitive, physical and social functioning, and increased levels of self-neglect, all of which may result in premature mortality (12). Despite this, to the best of our knowledge, there are no large population-based studies examining differences in aging on depression among older migrants and non-migrants in Europe countries.

Thus, the first aim was to analyze differences between migrants and non-migrants regarding socioeconomic status, depression, multimorbidity, healthy aging, and lifestyle behaviors. The second aim was to examine the impact of healthy aging on late-life depression among migrants and non-migrants using predominantly nationally representative, community-based data from European participants included in the Aging Trajectories of Health: Longitudinal Opportunities and Synergies (ATHLOS) cohort. Shedding light on migration and healthy aging is essential for promoting cross-cultural understanding of late-life depression in Europe.

## Materials and methods

### Study design and data extraction

The present study used data from the Aging Trajectories of Health: Longitudinal Opportunities and Synergies (ATHLOS) project (13). Longitudinal data from 17 international cohort studies related to health and aging were harmonized to obtain an integrated dataset and better understand aging and health processes. The Committee on the Ethics of Clinical Research approved the study protocol, CEIC Fundació Sant Joan de Déu (Protocol No: PIC-22-15). All data were anonymised, and EHR confidentially was respected following national and international law.

We selected the four studies of the ATHLOS project in which the variables of interest (those regarding migration and depression) were available. The following studies were included in the analyses: the English Longitudinal Study of Aging (ELSA) (14), the Collaborative Research on Aging in Europe (COURAGE in Europe) study (15), the Irish Longitudinal Study on Aging (TILDA) (16), and the Survey of Health, Aging and Retirement in Europe Study (SHARE) (17). These studies were performed in different countries. ELSA considered population from England, the COURAGE study covered Finland, Poland and Spain, TILDA included participants from Ireland, whereas the SHARE study comprised several European countries, such as the Czechia, Hungary, Poland, Denmark, Estonia, Finland, Ireland, Sweden, United Kingdom, Greece, Italy, Portugal, Slovenia, Spain, Croatia, Austria, Belgium, France, Germany, Luxembourg, Switzerland, and Netherlands.

It is worth mentioning that the baseline time of each participant differs. Thus, we did not use a fixed time-point. Instead, we used the first time each individual was included in the study as a baseline time. This setting increased the sample size. In addition, given that the ELSA, TILDA, and SHARE studies included participants aged 50 years or older and that the COURAGE study oversampled participants aged 50+ years, we decided to include participants from that age onward to allow comparability among studies. Moreover, we excluded those participants who participated *via* proxy due to cognitive problems or severe physical limitations, resulting in a final sample of 122,571 individuals.

### Variables

The following variables resulted from a stringent, *ex post* harmonization process using systematic harmonization methodology and tools from Maelstrom Research (13, 18).

#### Sociodemographic variables

We included sociodemographic variables such as age (years), gender (female/male), level of self-reported education (less than primary, primary, secondary, and tertiary), current marital status (single, married or currently cohabiting, separated or divorced, and widowed), and quintiles of household wealth (first quintile indicating lowest level). Moreover, we included dichotomized variables related to the occupation (current employment status and retirement). Additionally, we created a regional variable according to the United Nations Statistical Division (UNSD) regional classification (19), resulting in five regions: Eastern Europe (Czechia, Hungary, and Poland), Northern Europe (Denmark, Estonia, Finland, Ireland, Sweden, and United Kingdom), Southern Europe (Greece, Italy, Portugal, Slovenia, Spain, and Croatia), Western Europe (Austria, Belgium, France, Germany, Luxembourg, Switzerland, and Netherlands), Western Asia (Israel).

#### Late-life depression

Depression was assessed using different standardized tools in the included studies. The COURAGE study measured the presence of a 12-month depressive episode using an adapted version of the World Health Organization Composite International Diagnostic Interview (CIDI) (20). An algorithm based on ICD-11 depressive episode criteria (21) and the information provided by the participants regarding having received medication or psychological treatment for depression in the previous 12 months before baseline was used to determine a major depressive episode. ELSA used the 8-item version of the Center for Epidemiologic Studies Depression Scale (CES-D) (22). In this version, the response options are “yes” or “no” instead of the Likert-scale items from the 20-item CES-D scale. For each respondent, the total number of “yes” responses to questions 1, 2, 3, 5, 7, 8, and the “no” responses to questions 4 and 6 (reversed items) were summed to arrive at a total depressive symptom score ranging from 0 to 8. We classified those who reported four or more depressive symptoms as having significant depressive symptoms since this cut-off has been found to produce comparable results to the 16-symptom cut-off for the well-validated 20-item CES-D scale (23). In the case of TILDA, the 20-item CES-D scale was used (24). The CES-D is a 20-item measure assessing symptoms of depression with items phrased as self-statements, and respondents rate how frequently each item applied to them over the past week. In this version, the rating is based on a 4-point Likert scale ranging from 0 (”rarely or none of the time”) to 3 (”most or all of the time”). The responses are summed, ranging from 0 to 60, with higher scores indicating the presence of more symptomatology. A score of 16 or higher was proposed as an optimal cut-off score (24). Finally, SHARE used the EURO-D 12-item scale, developed and validated for the EURODEP studies to measure depressive symptoms across different European centers accounting for regional differences (25, 26). The EURO-D consists of 12 items scored by summing item scores for individual symptoms that are scored as 0 and 1 when they are “not present” and “present”, respectively. We selected four or more like a proper cut-off score, according to Prince et al. (26). The final harmonized variable was dichotomous (yes/no) and was created using the indicated cut-off score for each tool and population based on previous studies (20, 22–24, 26).

#### Migration

To allow comparability among studies, we used the migration status (migrant/non-migrant) and the number of years since migration—the number of years since migration was collected as a continuous variable. The included original studies did not provide any other variable related to the immigration process.

#### Somatic comorbidities

Non-communicable diseases (NCDs) and multimorbidity have been commonly reported as risk factors for worse mental health, showing a bidirectional relationship (27, 28). We considered the self-reported diagnosis of NCDs, which was common among studies: diabetes, hypertension, joint disorders (arthritis, rheumatism, or osteoarthritis), asthma, chronic obstructive pulmonary disease (COPD), coronary artery disease, and stroke. Multimorbidity was defined as having two or more of the abovementioned NCDs and depression.

#### Healthy aging scale

The healthy aging scale was explained in more detail elsewhere (29). Briefly, a healthy aging scale was developed by the ATHLOS project researchers using a worldwide cohort (13). This scale used items about intrinsic capacity and functional ability based on the WHO’s concept of healthy aging (30). The healthy aging scale covers different domains, such as vitality, sensory skills, locomotion/mobility, cognition, activities, and instrumental activities of daily living. Forty-one study-specific variables were harmonized into dichotomous items indicating the presence or absence of difficulties (29). The scale is a continuous variable distributed as a Normal with a mean of 50 and SD of 10, and higher scores mean better health status.

#### Lifestyles behaviors

Three different lifestyles and health behaviors were included: weekly frequency alcohol consumption (never, rare, and often), lifetime history of smoking (never smoker, ex-smoker, and current smoker), and physical activity (the practice of vigorous exercise during the last 2 weeks, coded as *yes* or *no*).

### Statistical analysis

Categorical variables were compared using the Chi-square test or Fisher’s exact test, and continuous variables were compared using the Mann-Whitney *U*-test to assess differences between migrant and no-migrant groups. Model selection was performed with Akaike Information Criterion (AIC), and a two-sided *p*-value of < 0.05 was considered for coefficients to be statistically significant. All modeling and computation of results were carried out using the statistical software R version 4.0.2 (31).

Prevalence rates and odds ratios (OR) for depression with 95% confidence intervals (CI) were calculated for all respondents *via* the fitting of multivariate logistic regression models. We applied the same models for all subjects in the sample. Depression was the dichotomous response variable, and the following set of predictors adjusted all models: age, marital status, level of education, living alone, employment, retirement, wealth, migration status, years since migration, region, health, aging status, multimorbidity, alcohol, and smoking.

## Results

### Descriptive analysis

The characteristics of the sample according to the migration status of the participants are presented in Table 1. A total of 122,571 individuals were included, of which 11,799 (9.60%) were migrants. Participants’ mean age was 63.6 (SD = 9.98), and there were no differences between migrant and non-migrant groups (63.81% vs. 63.55). In the overall sample, the percentage of females was 54.87%, slightly higher in the migrant group (55.57%) than in the non-migrant group (54.66%). More than half of the sample (51.75%) completed secondary education, and 20.51% reported having tertiary education. Although the migrant group reported having more proportion of participants with low primary levels of education (13.70%), a higher proportion of migrants had tertiary education (28.21%) compared to non-migrants (19.65%). Most of the samples (77.77%) were married, and 13.79% were widowed. The migrant group showed a lower percentage of single participants (4.71 vs. 6.65%) but a higher proportion of divorced people compared to the non-migrant group (9.76 vs. 7.82%). Only a few participants (19.66%) reported living alone, and the same percentage was found in both groups.

**TABLE 1:** Differences in socioeconomic status, healthy aging, multimorbidity, depression, and lifestyle behaviors between migrants and non-migrants.

Variables	Total sample (*n* = 122,571)	Non-migrants (*n* = 110,772)	Migrants (*n* = 11,799)	Effect size^a^	*P*-values^b^
**Age, mean (SD)**	63.6 (9.98)	63.55 (10.87)	63.81 (10.54)	−0.01	0.970
**Sex (female), n (%)**	77148 (54.87)	69067 (54.66)	8081 (55.57)	0.01	0.037
**Level of education, n (%)**				0.07	<0.001
Less than primary	12640 (9.19)	11387 (9.12)	1253 (8.84)		
Primary	25660 (18.55)	23720 (19.11)	1940 (13.70)		
Secondary	71609 (51.75)	64634 (52.01)	6975 (49.24)		
Tertiary	28391 (20.51)	24395 (19.65)	3996 (28.21)		
**Marital status, n (%)**				0.03	<0.001
Single	9074 (6.45)	8389 (6.65)	685 (4.71)		
Married	101068 (71.77)	90632 (71.80)	10436 (71.86)		
Divorced	11285 (7.99)	9867 (7.82)	1418 (9.76)		
Widowed	19328 (13.79)	17345 (13.74)	1983 (13.66)		
**Living alone (yes), n (%)**	28054 (19.66)	25146 (19.90)	2908 (19.99)	0.00	0.780
**Employed (yes), n (%)**	46286 (33.26)	41497 (33.09)	4789 (33.25)	0.00	0.700
**Retired (yes), n (%)**	66166 (47.47)	59603 (47.82)	6563 (45.61)	0.01	<0.001
**Wealth, quintiles, n (%)**				0.03	<0.001
1st (worse)	26626 (19.77)	23458 (19.41)	3168 (22.90)		
2nd	26449 (19.64)	23602 (19.53)	2847 (20.58)		
3rd	26366 (19.58)	23596 (19.52)	2770 (20.02)		
4th	27316 (20.29)	24768 (20.49)	2548 (18.42)		
5th (best)	27935 (20.73)	25432 (21.04)	2503 (18.09)		
**Region, n (%)**				0.30	<0.001
Southern Europe	25737 (21.00)	24563 (22.18)	1174 (9.95)		
Eastern Europe	15742 (12.85)	15233 (13.75)	509 (4.31)		
Northern Europe	37613 (30.70)	34224 (30.91)	3389 (28.72)		
Western Europe	40220 (32.82)	35385 (31.94)	4835 (40.98)		
Western Asia	3223 (2.63)	1334 (1.21)	1889 (16.02)		
**Healthy aging score, mean (SD)**	52.8 (9.43)	51.74 (9.67)	39.64 (18.24)	−0.07	<0.001
**Multimorbidity (yes), n (%)**	36167 (25.74)	31973 (25.38)	4194 (29.00)	0.03	<0.001
**Diseases (yes), n (%)**					
Diabetes	15164 (20.12)	13232 (19.52)	1932 (25.55)	0.05	<0.001
Hypertension	50951 (36.25)	45444 (36.08)	5507 (38.03)	0.01	<0.001
Joint disorders	33051 (23.52)	29644 (23.53)	3407 (23.53)	0.00	0.980
Asthma	6115 (5.20)	5441 (5.13)	674 (5.69)	0.01	0.009
COPD	7995 (5.71)	7105 (5.64)	890 (6.14)	0.05	0.013
Coronary artery disease	16859 (41.76)	14740 (41.86)	2119 (49.41)	0.01	<0.001
Stroke	5534 (3.98)	4828 (3.83)	706 (4.88)	0.02	<0.001
**Depression (yes), n (%)**	32095 (23.42)	27777 (22.57)	4318 (31.13)	0.06	<0.001
**Alcohol, n (%)**				0.08	
Never	40105 (29.19)	34713 (28.47)	5392 (38.32)		<0.001
Rare	56163 (40.89)	50658 (41.10)	5505 (39.12)		
Often	41042 (29.93)	37864 (30.73)	3175 (22.56)		
**Smoking, n (%)**				0.02	<0.001
Never smoked	70533 (50.61)	62921 (50.39)	7612 (53.10)		
Ex-smoker	41612 (29.90)	37671 (30.17)	3941 (27.49)		
Current smoker	27057 (19.49)	24274 (19.44)	2783 (19.41)		
**Physical activity (yes), n (%)**	70154 (50.08)	63063 (50.21)	7091 (49.15)	0.00	0.016

Household income was divided into five quintiles (the first indicating the lowest income). The marital status “married” category included “currently married or cohabiting,” and “divorced” included “divorced or separated.” COPD, chronic obstructive pulmonary disease.

^a^Based on Cohen’s *d* for numerical variables and Cramer’s V for categorical variables.

^b^Based on U Mann-Whitney tests for numerical variables and Chi-square tests for categorical variables.

Regarding employment, almost half of the sample was retired (47.47%), and the migrant group showed less proportion of retired participants (45.61 vs. 47.82%). Only 33.26% were active workers in the total sample, and no differences were found between groups. The proportion of household wealth in the total sample was equally distributed in five quintiles. Non-migrants showed higher proportions of participants classified in the fourth (20.49 vs. 18.42%) and fifth (21.04 vs. 18.09%) quintile of household wealth, indicating better income than migrants.

Regarding health variables, healthy aging was better in non-migrants (51.74; SD = 9.67) than in migrants (39.64; SD = 18.24). Similarly, migrants showed a higher prevalence of some of the included NCDs, such as diabetes (25.55%), hypertension (38.03%), asthma (5.69%), COPD (6.14%), coronary artery disease (49.41%), stroke (4.88%), as well as multimorbidity (29.00%). In addition, the migrant group reported less physical activity than the non-migrant group (49.15 vs. 50.21%). However, migrants showed lower current alcohol drinking (22.56 vs. 30.73%) and past smoking habits (27.49 vs. 30.17%) compared to non-migrants. Finally, migrants reported higher levels of depression than non-migrants (31.1 vs. 22.57%).

Table 2 shows the associations of risk and protective factors on depression in middle-aged and older adults. Regarding sociodemographic characteristics, gender (female), increased age, current marital status (divorced and widowed), occupation (current employment status), and higher education were also related to depression. Concerning different regions of the globe, older adults from Eastern and Northern Europe and Western Asia reported fewer depressive symptoms than those from Southern Europe. The migration status and increased years of migration in the host country were positively associated with depression in the total sample.

**TABLE 2:** Results from multivariate logistic regression analyses with late-life depression as an outcome variable in the total sample.

Variables	Total sample (*N* = 122,571)		
	OR	95% CI OR	*P*-value
**Sex**			
Female (ref.)	-	-	-
Male	−0.4765655	[−0.509 to −0.443]	<0.001
**Age**	1.0456273	[0.792 to 1.298]	<0.001
**Marital status**			
Single (ref.)	-	-	-
Married	−0.1104685	[−0.172 to −0.048]	<0.001
Divorced	0.2367125	[0.159 to 0.313]	<0.001
Widowed	0.0927354	[0.020 to 0.165]	0.012
**Level of education**			
Less than primary (ref.)	-	-	-
Primary	0.1848338	[0.127 to 0.242]	<0.001
Secondary	0.1262172	[0.071 to 0.180]	<0.001
Tertiary	0.0717273	[0.008 to 0.135]	0.027
**Employed**			
No (ref.)	-	-	-
Yes	−0.2070587	[−0.249 to −0.164]	<0.001
**Migration**			
No (ref.)	-	-	-
Yes	1.2311043	[0.914 to 1.547]	<0.001
**Years since migration**	0.0033060	[0.001 to– 0.005]	0.010
**Region**			
Southern Europe (ref.)	-	-	-
Eastern Europe	−0.3399909	[−0.392 to −0.287]	<0.001
Northern Europe	−0.5070727	[−0.551 to −0.462]	<0.001
Western Europe	0.0546353	[0.012 to 0.096]	0.010
Western Asia	0.0446375	[−0.052 to 0.141]	0.368
**Healthy aging score**	−0.1002261	[−0.102 to −0.098]	<0.001
**Multimorbidity**			
No (ref.)	-	-	-
Yes	0.2449217	[0.211 to 0.278]	<0.001
**Alcohol**			
Never	-	-	-
Rare	−0.2320182	[−0.268 to −0.195]	<0.001
Often	−0.3078021	[−0.348 to −0.266]	<0.001
**Smoking**			
Never smoked (ref.)	-	-	-
Ex-smoker	0.0497546	[0.013 to 0.086]	0.007
Current smoker	0.2037712	[0.162 to 0.244]	<0.001
**Year of birth**	0.0406845	[0.031 to 0.050]	<0.001
Age*year of birth	−0.0005509	[−0.001 to −0.000]	<0.001
Migration*healthy aging	−0.0198708	[−0.025 to −0.014]	<0.001

**FIGURE 1:**
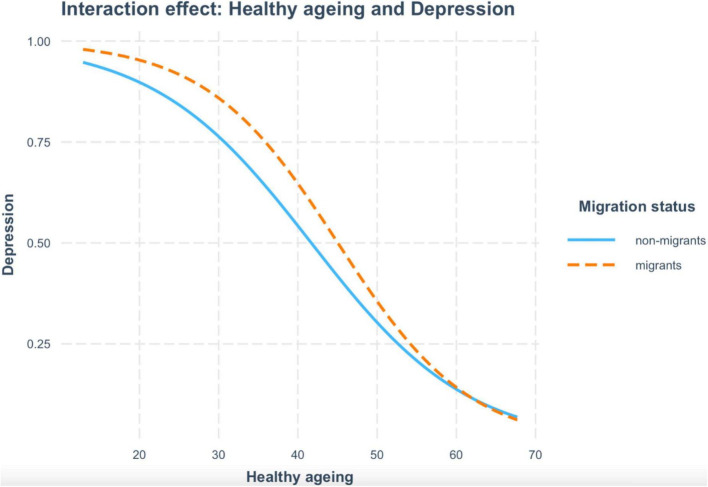
According to migration, the figure depicts the interaction between healthy aging and depression symptoms. The orange dashed line corresponds to migrants, and the blue line corresponds to non-migrants.

Regarding health variables, better healthy aging was associated with fewer depressive symptoms. Contrary, multimorbidity was associated with greater levels of depression. Similarly, past or present smoking habits were associated with higher depressive symptoms, whereas drinking alcohol regularly was associated with fewer depressive symptoms. In addition, the interaction between migration and healthy aging was significant. As shown in Figure 1, better healthy aging was associated with a lower risk of depressive symptoms in both groups. However, worse healthy aging was associated with a greater risk of depressive symptoms in migrants than in non-migrants.

## Discussion

The present work investigated risk and protective factors associated with late-life depression among migrants and non-migrants, using harmonized data from different population-based cohort studies (ELSA, COURAGE in Europe, TILDA, and SHARE) from 23 countries. In addition, the study examined for the first time the impact of healthy aging [measured by a common scale using harmonized data from 16 international aging cohorts (29)] on depression among middle-aged and older migrant and non-migrant populations. The findings contribute to our understanding of the interplay of migration and aging processes on depression in the elderly in Europe.

The descriptive analyses indicated some relevant differences between migrants and non-migrants in terms of socioeconomic and health status (disease prevalence, multimorbidity, healthy aging, and depression) that should be noteworthy. Specifically, migrants compared to their counterparts reported a lower household wealth as well as a higher prevalence of NCDs (except for joint disorders), multimorbidity, lower scores on the healthy aging scale (meaning worse functioning and intrinsic capacity), and also, increased levels of depression in older adults. Although we did not examine this association directly, previous research has shown a clear association between lower socioeconomic status and worse health status (32, 33). Notably, it has also been reported in the general population suggesting that better wealth is often associated with better healthcare access resulting in better disease management and improved health status (34). Nevertheless, studying socioeconomic factors and their relationship with health in the migrant population is quite cumbersome since several factors may play a role in its association. For example, certain occupations and precarious working conditions usually hamper the possibility of attending medical visits, impacting the individual’s health. Other authors emphasize the relevance of considering pre-migration and post-migration factors on the dynamics of their health, such as pre-migration socioeconomic status may impact health and well-being after migration (22).

On the contrary, a higher proportion of tertiary education was found in migrants (28.21%) compared to non-migrants (19.65%). Previous studies also reported higher education among migrants (6, 35, 36) than non-migrants, suggesting growth in highly skilled migration from low-and middle-income countries to high-income countries. However, high educational level among migrants has also been associated with common mental disorders, suggesting possible psychological distress due to a sense of unachieved goals across the lifespan (37). In this sense, it is essential to note that although factors involved in the aging process are broadly similar across populations, migrant-specific risk factors may also play a relevant role in impacting health and quality of life in the elderly (38). These include exposure to adverse events and health risks before, during, and after migration, such as low socioeconomic status, low level of acculturation, lack of social support, discrimination, and the length of migrants’ residence in the host country. It has been found that the lack of social support negatively impacts physical activity behaviors in culturally and linguistically diverse migrant groups. Notably, they often have to cope with more barriers and challenges in accessing healthcare or undertaking preventative measures to achieve good health. This data would support the urgent need for the development of culturally appropriate programs designed to impact health behaviors positively (39).

Concerning the impact of risk factors on late-life depression, as expected, increased age, gender (female), current marital status (divorced and widowed), and multimorbidity were related to depression (40). Previous research has extensively shown that older adults differ from younger populations mainly due to increased medical comorbidities and functional impairments (41). Furthermore, age-specific conditions and also psychosocial factors, such as life events (death of a loved one, socioeconomic difficulties), loss of status, or feeling of loneliness, contribute to a greater risk of depression (42, 43), particularly in females. On the contrary, some protective factors, such as being married and being employed, have also been associated with decreased late-life depression levels, suggesting that social support and keeping active may be particularly relevant in older adults.

Our results also showed that migration status and the increased number of years since migration in the host country were associated with greater levels of depression symptoms in middle-aged and older adults. Our findings concur with previous prospective studies showing an increased risk for depression between older migrants and non-migrants in Europe (6). Moreover, they are in line with the few longitudinal studies existing in the literature, reporting higher risks for worse health outcomes (i.e., physical functioning and depressive symptoms) for migrants compared to non-migrant populations (44). Notably, the interaction between migration and healthy aging status was significant, indicating that better healthy aging was associated with a lower risk of depressive symptoms in both groups. However, worse healthy aging was associated with a greater risk of depressive symptoms in migrants than in non-migrants. Some hypotheses have been postulated to explain the health disparities among older migrant and their non-migrant counterparts that also resonate with our results. For instance, the persistent inequality hypothesis raises that inequalities remain stable across the lifespan. Similarly, the cumulative disadvantage hypothesis indicates that the accumulation of socioeconomic difficulties leads to increased health risks and health inequalities between older migrants and non-migrants (45).

### Strengths and limitations

A major strength of this study is the use of a large, harmonized, and multiregional database. In this way, we gathered data from different cohort studies using equivalent variables and a considerable representation of migrants in our sample (*n* = 11,799). This translates into an advantage because migrants and ethnic minority populations are often excluded or underrepresented in epidemiological studies (46). Moreover, we considered the region of residence, including different European regions, such as Southern, Eastern, Northern, and Western Europe, and also Western Asia. Another strong point is the inclusion of the healthy aging scale in the analyses and the interaction between migration and health status. Previous studies of risk and protective factors in depression used single indicators of aging, such as cognitive functioning, morbidity, and functional impairment (6). Instead, the healthy aging scale is a composite measure of aging constituted by several items and domains, which could be affected over the lifespan and impact depression, providing us with a reliable, innovative and integrated measure of functioning across studies and populations.

Our findings should be considered in light of limitations. First, we used the harmonized depression variable that was built, taking into account the indicated cut-off scores for each tool and population. Notwithstanding, since this variable was created from three different tools with distinct periods (the last year in the CIDI, the last week in the CES-D, and the last month in the EURO-D), it could have introduced some bias. However, we consider that the impact would be minimal because the EURO-D was a derived measure from several scales, including the CES-D (47). Second, when performing the analyses, we excluded those participants with missing values in the variables of interest. As missingness could be influenced by the outcome (participants with depression may be more likely to refuse to answer questions related to mental health), we could not sustain that missing values were missing at random. For this reason, we did not conduct multiple imputations and went ahead with complete case analysis. Doing so could have influenced the results, underrepresenting those participants with depression. Third, the study of the risk and protective factors related to depression was constrained to the availability of variables in the harmonized studies. Future studies should analyze the impact of pre-migration and post-migration factors on depression. Notably, including individual characteristics, such as resilience and self-esteem, and social variables like feelings of loneliness, close relationships, and social participation, would have been enlightening as explanatory variables. On one side, high levels of resilience—psychosocial stress-resistance—are associated with a higher ability to cope with stressful situations during the migration process and, in turn, lead to better self-esteem (48, 49). Similarly, social support and a strong social network buffer the stressful effect of the migration process and affect mental health (50, 51). Moreover, there was a lack of information regarding migration as the reason for migration and the country of birth, which could have turned insights into this research. Consequently, our results may not be generalizable to other global areas, but our data support further research.

## Conclusion

In conclusion, healthy aging and migration are complex multidimensional processes that may be influenced at micro, meso, and macro levels over the life course (52). Thus, more studies focusing on socio-ecological models are needed to understand better the complex interplay of several determinants of health across the lifespan at the individual, interpersonal, and social/structural levels among migrant and non-migrant populations (53, 54). The diversity in individual characteristics, lifespan processes, and contextual factors shaping aging processes underscore a bio-psychological-spiritual comprehensive approach to policies, practices, and research in the field of healthy aging. This is also crucial to accommodate the new needs of the growing number of older migrants in Europe and face up inequities in health and well-being.

## Data availability statement

The datasets presented in this study can be found in online repositories. The names of the repository/repositories and accession number(s) can be found in the article/supplementary material.

## Ethics statement

The study protocol was approved by the Committee on the Ethics of Clinical Research, CEIC Fundació Sant Joan de Déu (Protocol No: PIC-22-15). All data were anonymised and EHR confidentially was respected in accordance with national and international law. The patients/participants provided their written informed consent to participate in this study.

## Author contributions

IM and PC-N wrote the first draft of the manuscript. AS-N and DF analyzed the data and wrote sections of the manuscript. JA-M, ML, BT-A, and SK made substantial contributions to the acquisition of data for the work and revised it critically for important intellectual content. All authors approved the final version and agreed to be accountable for all aspects of the work in ensuring that questions related to the accuracy or integrity of any part of the work are appropriately investigated and resolved.
